# A Systematic Review of the Electrodiagnostic Assessment of Neonatal Brachial Plexus

**DOI:** 10.31487/j.nnb.2020.02.12

**Published:** 2020-06-05

**Authors:** Virginia Orozco, Sriram Balasubramanian, Anita Singh

**Affiliations:** 1 Drexel University, School of Biomedical Engineering, Science and Health Systems, Philadelphia, Pennsylvania, USA; 2 Widener University, School of Engineering, Chester, Pennsylvania, USA

**Keywords:** neonate, brachial plexus, electrodiagnostic, nerve conduction studies, electromyography

## Abstract

Despite improvements in obstetric care, neonatal brachial plexus palsy continues to significantly impact infants’ lives worldwide, with an incidence of 1 to 4 per 1000 live births. While a majority of affected infants recover spontaneously by three months, 20-30% suffer permanent functional deficits that significantly impair their quality of life. Anatomical complexity of the brachial plexus results in varying degrees of injury and pathological changes at multiple levels within the plexus. Current clinical diagnosis relies on electrodiagnostic techniques such as nerve conduction (i.e., motor and sensory) and electromyography studies. These techniques not only aid clinicians to differentiate between axonal and demyelinating lesions, evident by changes in signal shape and conduction, but also provide prognostic information in cases of brachial plexus injuries. The presented study offers a comprehensive review of existing literature on electrodiagnostic techniques employed for assessing neonatal brachial plexus injuries.

## Introduction

Neonatal brachial plexus palsy (NBPP) continues to significantly impact infants’ lives, with a worldwide incidence of 1 to 4 per 1000 live births, despite improvements in obstetric care [1-6]. NBPP occurs due to over-stretching of the brachial plexus during birth, either by clinician applied (exogenous) or maternal (endogenous) forces [1, 5, 6]. Shoulder dystocia, a birthing scenario where the fetal shoulder/s impacts against the mother’s pubic symphysis, is strongly associated with NBPP [1-3, 5]. NBPP-related injuries can be avulsion of the nerve roots, over-stretching of the brachial plexus (BP) terminal nerves, or a combination [1, 5]. While 70-90% of NBPP cases have reported spontaneous recovery in the first three months of an infant’s life, 20-30% of the affected infants do not experience substantial spontaneous recovery, even by the third month [1, 7, 8]. Such cases result in a permanently reduced range of motion and a decrease in strength, size, and girth of the affected muscles [6, 9, 10]. A recent publication showed that the reported incidence of spontaneous recovery from NBPP is less than what has been previously hypothesized, thereby increasing the need for improved diagnostic tools [11].

Anatomical complexity of the BP offers challenges with localization of the injured site and is even more challenging when multiple sites are involved, which is often the case [12-17]. BP injury can either present as neuropraxia, axonotmesis, or neurotmesis, in addition to avulsion and neuroma in continuity [18-20]. Electrodiagnostic techniques, utilizing nerve conduction and electromyography studies, not only help provide information regarding the location of nerve injury but are also strongly correlated with the severity of injury [5, 12, 17, 20-23]. Axonal loss lesions (i.e., axonotmesis and neurotmesis) present with a reduction in the compound action potential (CAP) amplitudes and normal conduction velocities (CV) during nerve conduction studies, and fibrillation potentials and positive sharp waves during electromyography recordings [18, 20, 24]. Demyelinating nerve lesions show slow conduction and conduction block across the site of demyelination during nerve conduction studies [24]. Clearly, electrodiagnostic techniques serve as an invaluable tool for the diagnosis and prognosis of neonatal brachial plexus (NBP) injury by offering evidence about the location, severity, and type of nerve injury [12, 17, 19, 20, 23]. This paper offers a comprehensive review of current electrodiagnostic methods used to assess functional deficits and recovery in infants with neonatal brachial plexus injury.

## Methods

Clinical and experimental studies that use electrodiagnostic techniques, such as nerve conduction and electromyography studies, on normal and injured brachial plexus in neonate human and animal models were eligible for this review. The resulting publications based on a Boolean search of the PubMed database with the search keywords: neonatal, brachial plexus, electromyography, electrodiagnostic, nerve conduction, and injury; were compiled and thoroughly reviewed to be considered for this article.

### Findings

Although NBPP is clinically well-defined, very few electrophysiological studies on the neonatal brachial plexus, both normal and injured, have been published, as evidenced by the PubMed search results. A total of 14 articles resulted from using the search terms: neonate, brachial plexus, nerve conduction, and injury; of which eight were relevant, three had no abstract and were in a foreign language, and the remaining were irrelevant. The next search combination: neonate, brachial plexus, and electrodiagnostic, resulted in 10 relevant articles (out of the 12 results).

The final search combination: neonatal, brachial plexus, and electromyography yielded 88 publications, of which 42 were relevant, 14 had no abstract and were in a foreign language, and the remaining were irrelevant. After excluding duplicate studies and studies in a foreign language from the aforementioned relevant publications, a total of 39 relevant studies were used for this comprehensive review. 21 studies used electrophysiological techniques for NBPP diagnosis and prognosis in clinical scenarios [9, 17, 25-44]. Three articles utilized animal models for reporting electrophysiological parameters of avulsed or stretched BP [45-47]. The remaining 15 articles investigated the role of electrodiagnostic techniques in infants with BP injuries [1-5, 10, 12, 15, 17-20, 23, 24, 48].

### I Anatomy of the Brachial Plexus

**Figure 1:**
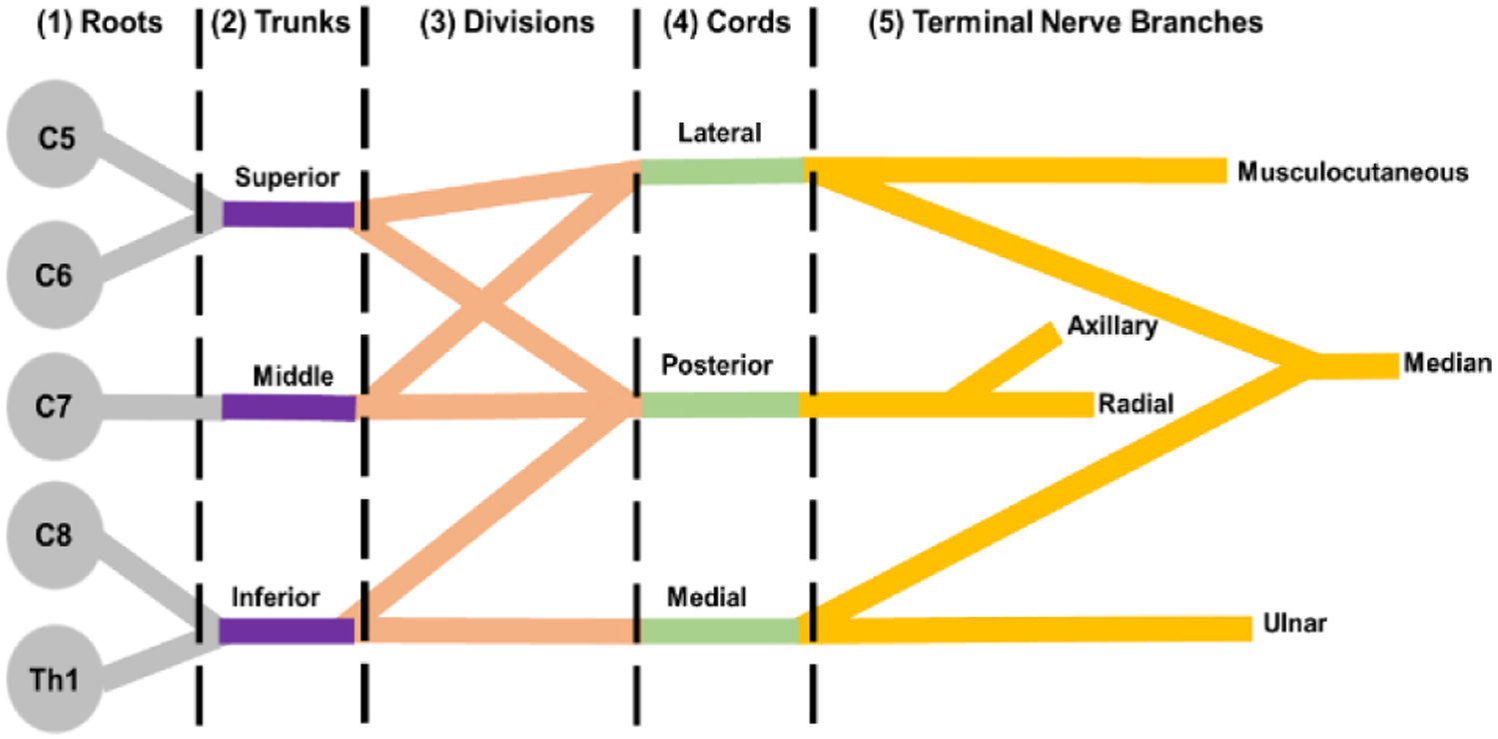
Schematic of the brachial plexus anatomy.

Brachial plexus is an intricate and complex network of nerves responsible for providing motor and sensory innervation to the right and left upper extremities [1, 8]. It originates as an extension from the ventral rami of C5 through Th1 spinal nerve roots on the sympathetic trunk and organized into five zones: (1) roots, (2) trunks, (3) divisions, (4) cords, and (5) terminal nerve branches, as shown in (Figure 1) [1, 8, 24, 49].

### II Classifications of Neonatal Brachial Plexus Injuries

Clinically, NBPP can be categorized by injury to any one of the spinal nerve roots (i.e., C5, C6, C7, C8, and Th1) and associated functional deficit of the affected limb. Table 1 shows the related functional deficit based on injury to the spinal nerve roots of the brachial plexus [1, 2, 5, 19, 20, 23, 48]. An example is where injury to the spinal nerve roots C5-C6 affects shoulder abduction and external rotation, elbow flexion, and wrist supination.

**Figure 2:**
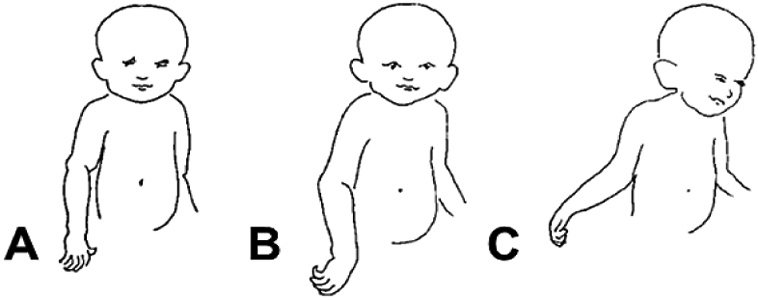
Representative images of the clinical appearance of neonatal brachial plexus palsy: **A)** mild, C5-C6 spinal nerve roots; **B)** intermediate, C5-C7 spinal nerve roots; **C)** severe, C5-Th1 spinal nerve roots (image adapted from [29]).

NBPPs have further been classified into four categories, referred to as the Narakas classification [1, 2, 48]. The four Narakas classification categories are (1) upper plexus palsy (i.e., Erb’s palsy, C5-C6 spinal nerve roots) and extended upper plexus palsy (i.e., C5-C7 spinal nerve roots), (2) intermediate plexus palsy (C7 and sometimes C8-Th1 spinal nerve roots), (3) lower plexus palsy (i.e., Klumpke’s palsy, C8-Th1 spinal nerve roots), and (4) total plexus palsy (C5-C8 and sometimes Th1 spinal nerve roots) [1, 2, 48]. Table 2 summarizes NBPP according to the Narakas classification, and (Figure 2) shows representative images of the clinical presentation of NBPP [1, 29, 48].

**Figure 3:**
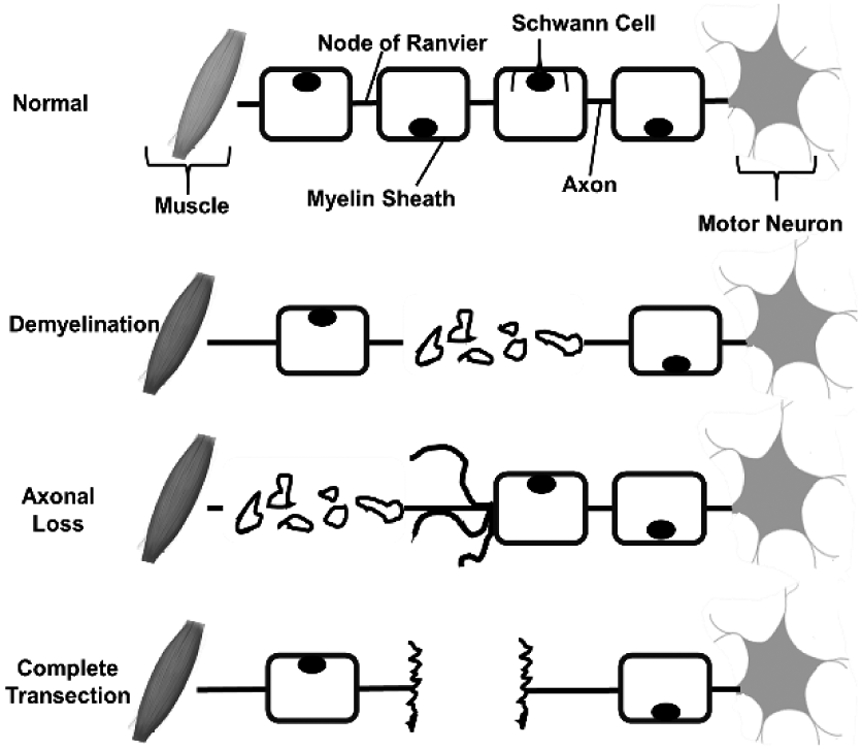
Classification of brachial plexus injury based on pathological outcomes (modified from [12]).

BP injuries can be further classified by pathological outcomes, such as neuropraxia, axonotmesis, and neurotmesis that describe axonal loss lesions, demyelinating lesions or a combination, respectively [12, 17, 20]. Neuropraxia lesions follow intact nerve fibers and damage to the myelin sheath. Axonotmesis observes axonal loss with the preservation of supporting connective tissue structures. Neurotmesis, the most severe outcome, is characterized by a complete transection of the axons and supporting connective tissue structures (Figure 3).

Infants who do not fully regain function after BP injury, not only have a limited range of motion and strength but also suffer further bony deformities and joint contractures [8, 23]. To avoid permanent damage and functional limitations of the affected upper extremity, surgeons have proposed the need for early surgical intervention [1, 3, 9, 11, 17, 23]. However, timing and type of surgery rely highly on early diagnosis and prognosis of the injury. Electrodiagnostic techniques enable the objective assessment of BP function, providing physicians with quantifiable measures of the extent of functional loss to predict the possibility of spontaneous recovery, if any [12, 17-19, 24]. These techniques include motor and sensory nerve conduction studies, needle electromyography, somatosensory-evoked potentials, and intraoperative evaluation [5, 12, 17-20, 22, 24]. This review article will focus only on the more commonly used techniques, namely motor and sensory nerve conduction and needle electromyography.

### III Using Electrodiagnostic Techniques to Assess Brachial Plexus Injury

Electrodiagnostic techniques, commonly employed to assess nerve response, include nerve conduction studies and electromyography (EMG). Nerve conduction studies allow the examination of the amplitude, conduction velocity, and latency of sensory and motor nerves [12, 17, 19, 24]. Sensory nerve conduction studies help distinguish if BP injuries are proximal or distal to the dorsal root ganglion [24]. Sensory nerve action potentials (SNAP) measure the extent of axonal loss through conduction velocity and amplitude [20]. Lesions proximal and distal to the dorsal root ganglion show intact SNAPs (i.e., preganglionic lesion) and impaired or absent SNAPs (i.e., postganglionic), respectively [24]. EMG records the electrical activity of motor fibers to detect signs of denervation and reinnervation [5, 12, 17]. Needle EMG studies quantify damaged axons as well as document the earliest signs of recovery by quantifying fibrillations and positive sharp waves [17]. Compound muscle action potentials (CMAP) represent the summation of motor units and is proportional to the amplitude [12]. A reduction or loss of CMAP amplitude indicates fewer or no motor neurons recruited, respectively, which help detect the extent of innervation to the muscle of interest [12, 17, 19]. Both nerve conduction and EMG techniques are often employed together to leverage the potential of electrodiagnostic techniques. Neuropraxia lesions show a reduced compound action potential (CAP) amplitude, and slow conduction velocity [12, 18]. Axonal loss during axonotmesis results in a reduction of CAPs while spontaneous activity of motor unit recruitment indicates nerve degeneration [12, 17, 18]. The most severe, neurotmesis, results in the absence of CAPs and motor unit activity [12, 17, 18]. In summary, characterizing electrophysiological parameters, such as conduction velocity, latency, amplitude, CMAP, and SNAP, help identify the type, location, and severity of brachial plexus injuries [12, 17, 24].

#### i Human Nerve Conduction Studies in Normal Uninjured Brachial Plexus

**Figure 4:**
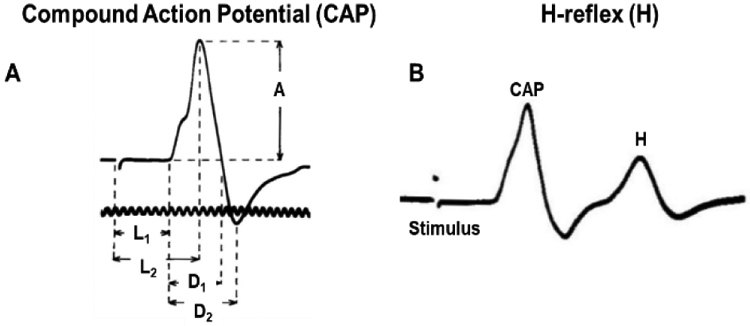
Schematic of electrophysiological response, **A)** Compound action potential (CAP) describing the parameters: amplitude (A), duration of the negative spike (D_1_), duration of the positive spike (D_2_), latency to the start of the potential from stimulus artifact (L_1_), and latency to peak potential (L_2_) and **B)** H-reflex, a late response measure when the stimulus directly stimulates the motor nerve fibers (adapted from [40]).

Thomas *et al.* (1960) performed nerve conduction studies in the uninjured ulnar nerve of 146 infants and children up to 14 years old. This early study examined the nerve conduction velocity and latency of H-reflex, as described in (Figure 4). The reported conduction velocity in infants (27.9 ± 0.47 m/s) was one-half of those previously reported in normal adults (47 to 73 m/s, ages 16-63 years) and reported an age-relationship of conduction velocity; such that the conduction velocity increased as the infants grew [40]. Several other nerve conduction studies also reported the age-relationship of conduction velocity in ulnar and median nerves (see Tables 3 & 4 for reported conduction velocities of ulnar and median nerves, respectively) [27, 28, 30, 33, 35, 41]. Gamstrop *et al.* (1963) performed ulnar and median nerve conduction studies in 86 infants and children up to 16 years old. The conduction velocity of the ulnar and median nerves of neonates (ulnar: 32.2 ± 4.4 m/s and median: 29.0 ± 3.7 m/s) was half of those in adolescents (16 years, ulnar: 67.6 ± 1.2 m/s and median: 63.6 ± 1.3 m/s) [27]. The study further reported the maturation rate of ulnar and median nerve conduction velocity. In the first three years of life, the ulnar nerve conduction velocity increased rapidly, while the median nerve conduction velocity slowly increased in the first year of life with a rapid increase in the adolescent years [27].

These findings demonstrate the distinct neurophysiological response characteristics of the brachial plexus terminal nerve branches, suggesting their potential for differentiating the type, location, and severity of NBP injury. Moglia *et al.* (1989) examined motor nerve conduction of intact ulnar and median nerves in 635 infants and children up to 12 years old and also reported an increase in conduction velocities with age [33]. Tiwari *et al.* (1996) reported the relationship between age and nerve conduction velocity and latency.

Motor-sensory nerve conduction studies on healthy median nerves from neonates (1-28 days) and infants (1 month-1 year) revealed that as age increased, nerve conduction velocity increased, and latency of H-reflex decreased [41]. Garcia *et al.* (2000) investigated the evolution of nerve conduction in the upper and lower limbs during the first year of life using motor-sensory nerve conduction studies. The study included 92 healthy infants and children aged one week to 6 years. Motor-sensory nerve conduction studies investigated motor-sensory conduction velocity, latency, and F-waves of the median, ulnar, peroneal, and tibial nerves [28]. While this study helped provide baseline electrophysiological parameters for brachial plexus responses in normal neonates and children, it also reported motor-sensory conduction velocities for neonates to be one-half of those previously reported in normal young adults. This finding is similar to those reported previously by Thomas *et al.* (1960) and Gamstorp *et al.* (1963) [27, 28, 40].

**Figure 5:**
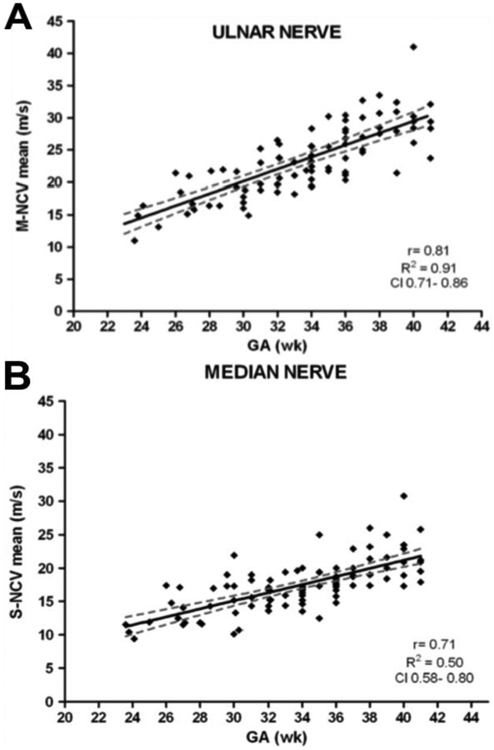
Linear correlation of **A)** ulnar nerve motor nerve conduction velocity (r = 0.80, R^2^ = 0.91, CI = 0.71-0.84) and **B)** median nerve sensory nerve conduction velocity (r = 0.71, R^2^ = 0.50, CI = 0.58-0.80) with gestational age (adapted from) [30].

More recent studies by Lori *et al.* (2018) and Ryan *et al.* (2019) examined the evolution of sensory-motor nerve conduction parameters in healthy pre-term and full-term infants, and in healthy neonates and adolescents, respectively. Lori *et al.* (2018) reported sensory-motor nerve conduction velocity (Figure 5), latencies of compound action potentials, sensory action potentials, and F-waves to have linear relationships with gestational age [30]. Ryan *et al.* (2019) further strengthened the previously reported findings on the linear relationship between nerve conduction velocity and age, based on data from 1849 healthy subjects (0-18 years) – the largest sample studied to date [35].

#### ii Human Nerve Conduction Studies in Injured Brachial Plexus

Kwast *et al.* (1989) conducted median and ulnar nerve conduction studies on 24 infants and children up to 15 years diagnosed with NBPP to assess how the injured neonate brachial plexus matures. The ability of the injured neonate BP to regenerate was better described by latency than conduction velocity [44].

**Figure 6:**
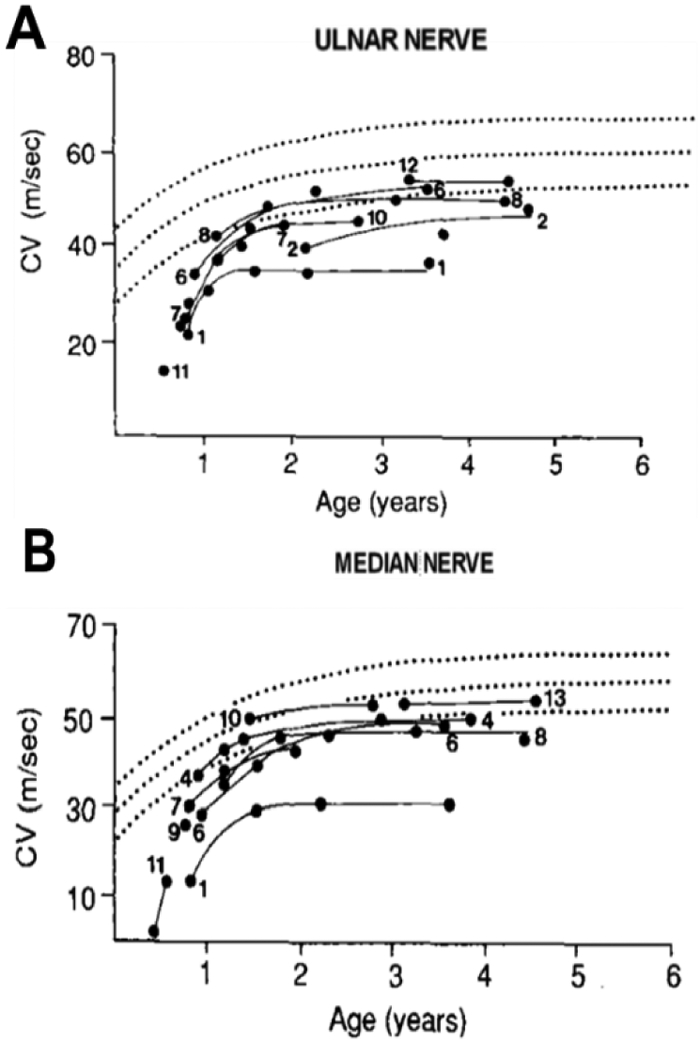
Age-relationship of conduction velocity in injured brachial plexus. **A)** ulnar nerve conduction velocity and **B)** median nerve sensory nerve conduction velocity (adapted from [44]).

The latency of the median nerve reached normal range by three years old, while only half of the ulnar latencies were within the normal range [44]. By three years old, however, conduction velocity did not differentiate regeneration ability as a function of maturation [44]. Similar to the nerve conduction studies performed on normal brachial plexus, Kwast *et al.* (1989) showed that conduction velocity in injured BP also increased as a function of age, as shown in (Figure 6) [27, 28, 30, 33, 35, 40, 41].

Heise *et al.* (2009) performed motor nerve conduction studies on the five terminal nerve branches of the BP (i.e., axillary, musculocutaneous, proximal/distal radial, median, and ulnar) in 54 infants with unilateral NBPP [9]. The study reported motor nerve conduction to be significantly different among all terminal nerve branches as early as ten days after birth, except in the median nerve [9]. Current clinical care for BP injuries highly relies on spontaneous recovery and a wait of three months before employing surgical interventions [1, 3, 50]. Early diagnosis, as reported in Heise *et al.* (2009), can significantly help with employing early surgical intervention approaches to improve outcomes in cases that offer less promise for spontaneous recovery.

#### iii Human Electromyography Studies in Injured Brachial Plexus

**Figure 7:**
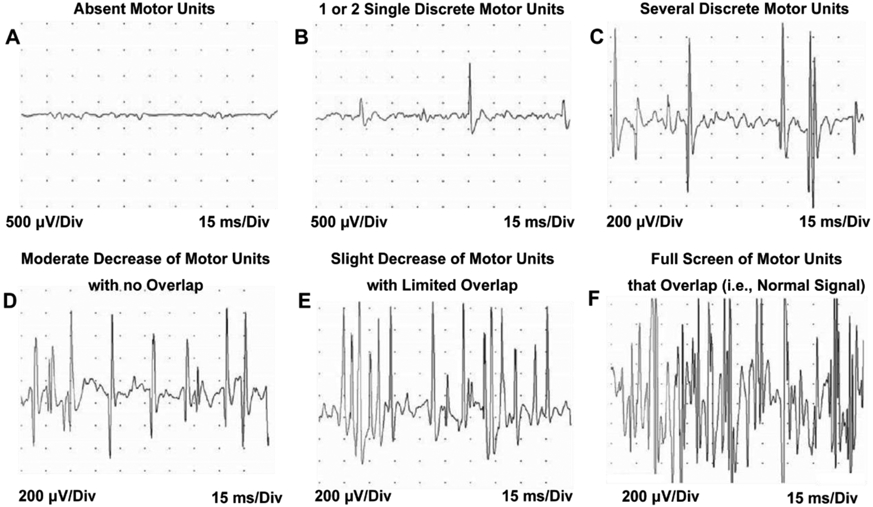
Representative neonate electromyography recordings used to identify brachial plexus injury type by examining motor unit action potentials (adapted from [39]).

The following studies used electromyography (EMG) to assess NBPP in infants. Talbert *et al.* (2011) reported the use of EMG to classify the prognosis in subjects with NBPP correctly. The EMG of the infraspinatus and latissimus dorsi muscles of 74 subjects (mean age: 5 years, age range: one month-13 years) to examine and rank the recruitment pattern of available motor units, as shown in (Figure 7) [39]. The ranked motor recruitment pattern was compared to the subjects’ Mallet Score (i.e., the assessment of active motion of the upper extremity) to assess the reliability of EMG in identifying the type of brachial plexus palsy. The authors found a significant correlation between the EMG of the infraspinatus muscle when dichotomized, and the Mallet Score to moderately classify neonatal brachial plexus prognosis [39].

Lindell-Iwan *et al.* (1995) performed EMG studies on deltoid, biceps, triceps, and infraspinatus muscles of 46 children (3 weeks to 7 months old) diagnosed with varying degrees of NBPP. This study evaluated the reliability of EMG to distinguish between injured BP nerve roots and predict prognosis. EMG testing was completed at 3-6 weeks (i.e., first visit), then at 6-28 weeks (i.e., second visit), and findings were compared to the subjects’ final clinical visit (<12 months from first EMG visit). Of the 46 children, 23 suffered C5-C6 nerve root injury that progressed to normal function at least twelve months from the first EMG test. Their EMG recordings at their first visit showed moderate damage that improved to a normal response at their second visit [29]. Of the fourteen children with C5-C7 nerve root injury, six progressed to normal function (6/14, ~43%), six to mild function (6/14, ~43%), and two to severe functional deficit (2/14, 14%). EMG also supported the prognosis as intermediate/severe functional deficit at the first visit, then progressed to mild/intermediate function and, in some cases, to normal function at their second visit [29]. The remaining children had a severe injury, C5-Th1 nerve root lesion, of which five progressed to severe functional deficit (5/9, 55%), and four progressed to mild/intermediate function (4/9, 46%) [29]. At the first visit, EMG showed severe functional deficit that progressed to mild/intermediate function; however, the final clinical appearance in these children was poor [29].

As a result, Lindell-Iwan *et al.* (1995) suggested that subjects with C5-Th1 nerve root injury would have benefited from microsurgical nerve repair [29]. EMG was shown to predict the prognosis of upper (i.e., C5-C6) and intermediate (C5-C7) plexus lesions appropriately, while in severe (i.e., C5-Th1) plexus lesions EMG predicted optimistic outcomes although the children experienced poor outcomes. Based on their findings, the authors suggest that newborns with NBPP would benefit from EMG studies at three weeks and again at 2-3 months to aid in determining the need for surgical intervention [29]. Paradiso *et al.* (1997) performed EMG in 78 infants with upper trunk NBPP (i.e., Erb’s palsy) [34]. This study reported denervation activity as early as day ten and up to day 60, as well as motor unit potential changes beginning at day 30 [34].

Yilmaz *et al.* (1999) performed needle EMG on day 27, day 50, and three months on 13 infants with neonatal brachial plexus injury [43]. Eight infants had an upper BP injury (i.e., Erb’s palsy), and five infants had total BP injury [43]. The functional outcome at twelve months was compared to the EMG readings from day 27, day 50, and three months. The EMG response of the eight infants with Erb’s palsy predicted good recovery, which was the last status of these children at twelve months [43]. In the five infants with total BP palsy, EMG predicted poor prognosis for four of them and a good prognosis for one, which was accurate except in the one infant where EMG suggested a good prognosis [43]. This study showed how EMG acquired at different times could predict the prognosis of upper and total brachial plexus palsies.

The following two studies performed needle EMG at week one, and months one and three in infants with NBPP to determine the best timing to predict prognosis. Malessy *et al.* (2011) performed needle EMG on 48 infants with only upper BP palsy to characterize the injury by quantifying the presence of spontaneous EMG activity and the absence of motor unit potentials [32]. At month one, the lack of motor unit potentials better predicted the severity of NBPP (82.9 ± 4.6%) compared to the presence of spontaneous EMG activity (26.1 ± 7.0%) [32]. These results, in combination with joint movement (out of the scope of this review), were then used in two groups of infants with NBPP to validate the reliability to predict the severity of varying degrees of NBPP. In the first group of 60 infants (mean age 31 days) with NBPP, the correctly predicted outcomes were 88.3% (53/60) [32].

In the second group of thirteen infants (mean age 31 days) with NBPP, the correctly predicted outcomes were 84.6% (11/13) [32]. This study showed that needle EMG at month one in comparison to the standard-of-care of three months seemed to be a better indicator of prognosis and to aid in planning surgical intervention that can minimize denervation [32, 50]. Van Dijk *et al.* (2012) also performed needle EMG at week one and months one and three in infants with NBPP to identify which time would best predict prognosis from elbow flexion [42].

Although most infants spontaneously recovered by three months, infants who did not recover were referred for surgical intervention to improve the function of the affected limb [42, 50]. In summary, needle EMG studies at one month could predict paralysis, suggesting that this technique can be used to aid clinician’s decision of early referral of infants to specialists for improved prognosis [29, 32, 42, 50].

#### iv Combined Nerve Conduction and Electromyography Human Studies in Injured Brachial Plexus

The following studies investigated the predictive reliability of EMG, combined with nerve conduction studies, to identify the severity and outcome of NBPP. Scarfone *et al.* (1999) performed EMG on the biceps and thenar muscles, as well as sensory nerve conduction studies on the radial, median, and ulnar nerves in 18 subjects (10 days-35 years) with NBPP [36]. The subjects' unaffected limb and healthy subjects were both used as controls to compare any changes with the affected limb responses [36].

The study reported a decrease in both motor units and M-wave amplitudes of both muscles and a reduction in SNAP amplitudes and latency of the nerves in the affected limb as compared to their unaffected limb and controls [36]. Brown *et al.* (2000) performed EMG on 16 subjects (4-14 years) with NBPP and age-matched healthy subjects [25]. The authors aimed to use EMG as a tool to evaluate the extent of functional loss experienced by children with BP injuries. Using the M-wave measure, Brown *et al.* (2000) reported muscle weakness was not only specific to denervation, but also to the limited number of recruited motor units [25].

Louis *et al.* (2010) reported the use of EMG and nerve conduction studies to assess the function of a 25-day-old neonate’s upper limb after presenting with the clinical appearance of Erb’s palsy (i.e., C5-C6 plexus lesion) [31]. The reported presence of denervation potentials from the deltoid EMG and normal ulnar and median nerve conductions were indicative of an upper brachial plexus palsy [31]. Estienne *et al.* (2005) showed that combined EMG and nerve conduction studies were able to identify BP involvement at day 23 [26].

#### v Animal Nerve Conduction Studies in Injured Brachial Plexus

Gonik *et al.* (1998) reported the length of the distal nerve segment and timing of EMG correlated with signs of denervation using a piglet animal model [45]. The study also investigated differences in EMG responses in adult pig and piglet animal models post-BP transection injuries using five healthy 2-day-old domestic piglets and two 6-month-old adult female pigs, respectively [45].

**Figure 8:**
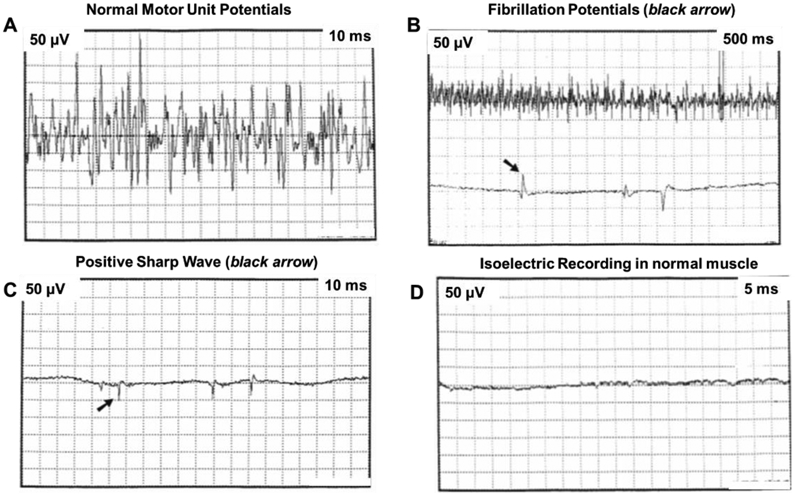
Representative electrophysiological recordings in a 2-day old piglet of axonal denervation (adapted from [45]).

The anesthetized piglets and pigs were subjected to transection of the C6-C8 and Th1 nerve roots to simulate the most severe avulsion-type injury associated with NBPP [45]. Muscle fibrillations between 24 and 48 hours after inducing damage were noted in the neonate piglets (Figure 8) as opposed to adult pigs, where denervation was demonstrated at day five after nerve root transection [45]. The findings from this animal study show similar results reported previously in humans that found differences in electrophysiological responses of injured BP in neonates versus adults [9, 44].

In another study, Takai *et al.* (2002) examined the electrophysiological response of the lower trunk BP using Japanese white rabbits [46]. In this study, the lower trunk of BP was stretched, and EMG was used to assess the extent of functional deficit. EMG reported conduction block due to neuropraxia, as histological studies observed intact but rearranged axons [46].

### IV Using Electrodiagnostic Techniques to Understand Recovery/Adaptation Post-Brachial Plexus Injury

#### i Human Studies Investigating Recovery Post Brachial Plexus Injury

Unlike a mature nervous system, the neonatal nervous system undergoes adaptation at both spinal and supraspinal levels to overcome the initial motoneuron loss resulting from NBPP [51]. Estienne *et al.* (2005) performed nerve conduction and EMG studies at different times on an infant with bilateral upper trunk BP injury [26]. At three months, the infant showed signs of regaining his reflexes in both limbs, which was suggestive of spinal cord adaptation [26].

#### ii Animal Studies Investigating Recovery Post Brachial Plexus Injury

Korak *et al.* (2004) performed NBP electrophysiological studies to investigate changes in the BP responses post-injury using a small animal model [47]. The study hypothesized that injury to the NBP complex would lead to permanent changes in a normal spinal cord architecture [47]. Neonatal (n = 15) and adult (n = 10) rats were subjected to crush injury at C5 and C6 levels (i.e., an upper BP complex injury). Functional muscle testing 12 weeks post-injury was performed on the musculocutaneous nerve that innervates the biceps muscle. After 12 weeks, the functional assessment showed axons originating from the nerve roots C5 and C6 had degenerated in both neonate and adult rats [47].

**Figure 9:**
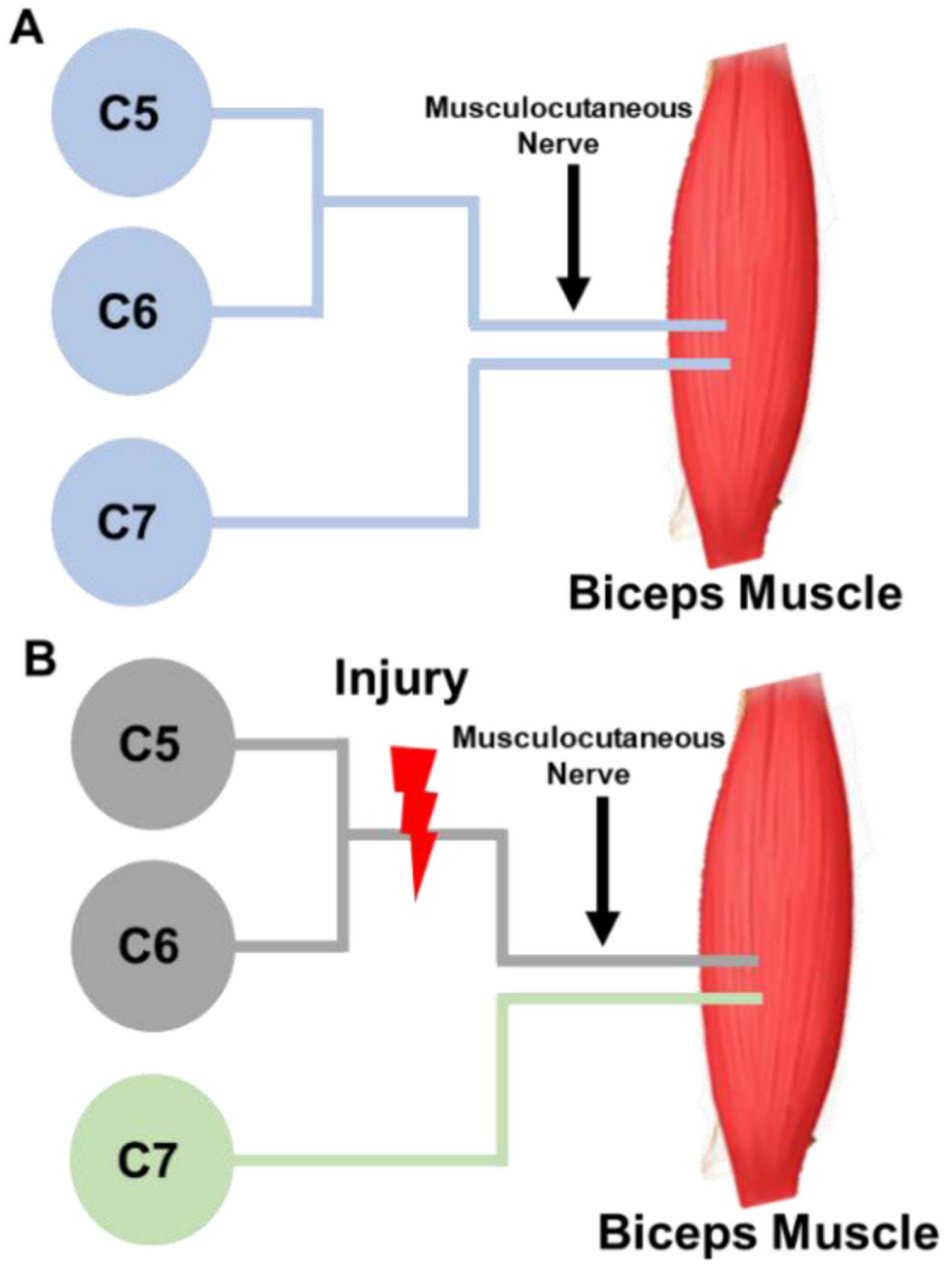
Schematic of C5, C6, and C7 nerve roots (i.e., upper brachial plexus) innervation to the biceps muscle before and after injury of the neonate. **A)** Schematic showing the involvement of all the nerve roots (blue) to the biceps muscle at birth. **B)** Schematic showing damage to C5-C6 (red thunderbolt) nerve roots leads to zero to limited involvement of the C5-C6 motoneuron to the biceps muscle (grey) and central adaptation from C7 motoneuron pool (green) to the muscle biceps (modified from [47]).

This study further showed recovery differences between neonate and adult rats by exploring the reinnervation of the C7 nerve root to the bicep muscles. The C7 motoneuron pool has been shown to have a link to the bicep muscle at the time of birth and disappear at normal maturation [47]. Because of the anatomical complexity of the BP, the authors resected the C5-C6 nerve roots to restrict electrical stimulation to C7 [47]. Neonates demonstrated central adaptation as C7 reinnervated the biceps muscle, as seen in (Figure 9), whereas adults showed minimal C7 contribution since at full maturation innervation of the biceps muscle is specific to C5 and C6 [47]. To further confirm spinal cord adaption differences in neonates and adults, retrograde labeling was used to quantify the contribution of C7 motoneuron [47]. The findings showed a significantly higher C7 motoneuron contribution after NBP injury compared to adults and controls [47].

Through these studies, it is evident that central nervous system adaptation occurs in severe cases of NBP injuries. Electrodiagnostic techniques can further help with early diagnosis of severe cases of BP injury to guide interventions that take advantage of the compensatory mechanisms of the central nervous system while avoiding maladaptive motor programming that occur as a result of poor prognosis [25].

### V Reliability Studies

Spires *et al.* (2017) examined the inter-rater reliability of interpreting electrodiagnostic results of subjects with NBPP [38]. Two board-certified reviewers reviewed electrodiagnostic data from 37 infants with varying degrees of NBPP to independently identify the type of palsy from the injured nerve roots [38]. The reviewers were able to agree on injury assessment for C5 (38%), C6 (78%), C7 (92%), C8 (81%), T1 (84%), and all (75%) nerve roots, thereby supporting a high inter-rater reliability assessment of nerve root lesions of NBPP [38].

Smith *et al.* (2018) compared electrodiagnostic studies and imaging to identify which modality identified the injury pathology of 54 infants with NBPP [37]. Imaging studies detected avulsion type injury for 69% cases, while electrodiagnostic studies detected it for 74% cases [37]. Electrodiagnostic studies had a specificity of 90% versus 70% for imaging studies, showing that electrodiagnostic studies in infants with NBPP could better identify the injury pathology [37].

## Conclusion

Electrodiagnostic techniques, currently employed in clinical scenarios, offer an objective and quantitative evaluation to distinguish lesion type and severity of BP injury [12]. Published nerve conduction studies have established relationships between age and conduction velocity. Furthermore, abnormal nerve signals, (acquired through conduction and EMG studies) observed as early as 10 days post-BP injury, can serve as a good predictor of injury prognosis. Such available data are critical in reforming current standard-of-care that hinders early intervention through heavy reliance on spontaneous recovery. Continued investigational studies utilizing electrodiagnostic techniques can continue to help better understand injury outcomes, direct improvements in existing diagnostic tools that offer better prognosis of BP injury, and advance the science of neonatal care.

## Figures and Tables

**Table 1:** Spinal nerve roots and related upper extremity function [1].

Function	Brachial Plexus (BP) Spinal Nerve Root	
Shoulder	Abduction - external rotation	Adduction - internal rotation
	C5, C6	C5-Th1
Elbow	Flexion	Extension
	C5, C6	C6, C7, C8
Wrist	Supination	Extension
	C5, C6	C5, C6, C7
	Radial inclination	Flexion
	C5, C6, C7	C6, C7, C8
	Pronation	Ulnar inclination
	C6, Th1	C7, C8
Hand	Extrinsic muscles	Intrinsic muscles
	C7, C8, Th1	C8

**Table 2:** Narakas classification of neonatal brachial plexus palsy [1, 2, 48].

Narakas Classification	Anatomical Location	Functional Deficit
Group I	C5-C6	Shoulder abduction, external rotation, elbow flexion, forearm supination
Group II	C5-C7	As above, plus wrist and digital extension
Group III	C5-T1	Flail extremity
Group IV	C5-T1	Flail extremity with Horner’s syndrome

**Table 3:** Ulnar nerve conduction velocity in normal brachial plexus [27, 28, 30, 33, 35, 40, 41].

**Thomas *et al*. (1960)**	Age*	**1 - 46d** **(N = 6)**									
	CV [m/s]	20.7 (0.7)									
**Gamstrop *et al*. (1960)**	Age**	**Birth - 1wk** **(N = 30)**	**1wk - 4mo** **(N = 18)**	**4mo - 1yr** **(N = 25)**	**1 - 3 yr** **(N = 21)**	**3 - 8yr** **(N = 26)**	**8 - 16yr** **(N = 26)**				
	CV [m/s]	32.2 (4.4)	42.6 (8.5)	49.9 (6.8)	59.8 (8.1)	65.4 (8.5)	67.6 (6.0)				
**Moglia *et al*. (1989)**	Age	**0 - 1yr** **(N = 9)**	**1 - 3yr** **(N = 27)**	**3 - 6yr** **(N = 24)**	**6 - 12yr** **(N = 33)**						
	CV [m/s]	48.2 (3.2)	57.2 (5.5)	56.4 (7.6)	57.9 (9.6)						
**Tiwari *et al*. (1996)**	Age***	**1 - 28d** **(N = 20)**	**2 - 12mo** **(N = 20)**								
	CV [m/s]	25.2 (2.5)	34.4 (6.0)								
**Garcia *et al*. (2000)**	Age	<**1mo** **(N = 11)**	**1 - 6mo** **(N = 12)**	**6 - 12mo** **(N = 12)**	**12 - 24mo** **(N = 15)**	**24 - 48mo** **(N = 17)**	**48 - 72mo** **(N = 17)**				
	CV [m/s]	25.0 (2.7)	36.3 (3.7)	45.0 (2.9)	48.9 (2.5)	54.2 (3.5)	56.5 (3.2)				
**Lori *et al*. (2018)**	Age	**23 - 25wk** **(N = 4)**	**26 - 27wk** **(N = 7)**	**28 - 29wk** **(N = 6)**	**30 - 31wk** **(N = 11)**	**32 - 33wk** **(N = 11)**	**34 - 35wk** **(N = 15)**	**36 - 37wk** **(N = 16)**	**38 - 39wk** **(N = 9)**	**40 - 41wk** **(N = 10)**	
	CV [m/s]	13.9 (2.3)	17.8 (2.5)	19.5 (2.6)	19.2 (3.3)	21.9 (2.8)	23.6 (3.1)	26.6 (3.7)	29 (3.5)	29.7 (4.6)	
**Ryan *et al*. (2019)**	Age	**0 - <1mo** **(N = 7)**	**1 - <6mo** **(N = 13)**	**6 - <12mo** **(N = 29)**	**12 - <24mo** **(N = 40)**	**2 - <3yr** **(N = 36)**	**3 - <4yr** **(N = 33)**	**4 - <5yr** **(N = 27)**	**5 - <10yr** **(N = 143)**	**10 - <15yr** **(N = 258)**	**15 - <18yr** **(N = 509)**
	CV [m/s]	35.0 (7.0)	43.0 (7.0)	51.0 (7.0)	53.0 (7.0)	56 (6.0)	58.0 (6.0)	60.0 (6.0)	61.0 (6.0)	62.0 (5.0)	63.0 (5.0)

N = number of observations; mean (standard deviation); d: day; wk: week; yr: year.

* 1 - 46d: pre-term infants.

** Birth - 1wk: Neonate; 1wk - 4mo: Early Infancy; 4mo - 1yr: Late Infancy; 1 - 3yr: Early Childhood; 3 - 8yr: Late Childhood; 8 - 16yr: Adolescence.

*** 1 - 28d: Neonate; 2 - 12mo: Infant.

**Table 4:** Median nerve conduction velocity in normal brachial plexus [27, 28, 30, 33, 35, 41].

**Gamstrop *et al*. (1960)**	Age*	**Birth - 1wk** **(N = 30)**	**1wk - 4mo** **(N = 18)**	**4mo - 1yr** **(N = 25)**	**1 - 3 yr** **(N = 21)**	**3 - 8yr** **(N = 26)**	**8 - 16yr** **(N = 26)**			
	CV [m/s]	29.0 (3.7)	33.9 (8.7)	40.0 (5.3)	49.5 (1.3)	58.3 (5.9)	63.9 (5.7)			
**Moglia *et al*. (1989)**	Age	**0 - 1yr** **(N = 9)**	**1 - 3yrs** **(N = 27)**	**3 - 6yrs** **(N = 24)**	**6 - 12yrs** **(N = 33)**					
	CV [m/s]	47.2 (2.8)	54.4 (6.4)	59.9 (8.6)	58.9 (9.8)					
**Tiwari *et al*. (1996)**	Age**	**1 - 28d** **(N = 20)**	**2 - 12mo** **(N = 20)**							
	CV [m/s]	26.6 (3.3)	36.6 (6.2)							
**Garcia *et al*. (2000)**	Age	<**1mo** **(N = 11)**	**1 - 6mo** **(N = 12)**	**6 - 12mo** **(N = 12)**	**12 - 24mo** **(N = 15)**	**24 - 48mo** **(N = 17)**	**48 - 72mo** **(N = 17)**			
	CV [m/s]	26.2 (2.2)	36.4 (3.7)	43.9 (3.4)	47.8 (2.3)	52.7 (3.7)	56.4 (2.4)			
**Lori *et al*. (2018)**	Age	**23 - 25wk** **(N = 4)**	**26 - 27wk** **(N = 7)**	**28 - 29wk** **(N = 6)**	**30 - 31wk** **(N = 11)**	**32 - 33wk** **(N = 11)**	**34 - 35wk** **(N = 15)**	**36 - 37wk** **(N = 16)**	**38 - 39wk** **(N = 9)**	**40 - 41wk** **(N = 10)**
	CV [m/s]	10.8 (1.1)	14.2 (2.4)	15.2 (3.1)	15.6 (3.7)	16.4 (3.0)	16.9 (3.0)	18.3 (1.9)	21.3 (3.0)	21.9 (4.1)
**Ryan *et al*. (2019)**	Age	**0 - <1mo** **(N = 5)**	**1 - <6mo** **(N = 14)**	**6 - <12mo** **(N = 12)**	**12 - <24mo** **(N =17)**	**2 - <5yr** **(N = 17)**	**5 - <10yr** **(N = 32)**	**10 - <15yr** **(N = 77)**	**15 - <18yr** **(N = 239)**	
	CV [m/s]	25.0 (3.0)	37.0 (9.0)	45.0 (13.0)	47.0 (5.0)	51.0 (6.0)	56.0 (7.0)	58.0 (4.0)	59.0 (3.0)	

N = number of observations; mean (standard deviation); d: day; wk: week; yr: year

* Birth - 1wk: Neonate; 1wk - 4mo: Early Infancy; 4mo - 1yr: Late Infancy; 1 - 3yr: Early Childhood; 3 - 8yr: Late Childhood; 8 - 16yr: Adolescence

** 1 - 28d: Neonate; 2 - 12mo: Infant
